# Grammatical Spelling and Written Syntactic Awareness in Children With and Without Dyslexia

**DOI:** 10.3389/fpsyg.2020.01524

**Published:** 2020-07-22

**Authors:** Marie Van Reybroeck

**Affiliations:** Delilab, Psychological Sciences Research Institute (IPSY), UCLouvain, Louvain-la-Neuve, Belgium

**Keywords:** dyslexia, grammatical spelling, written syntactic awareness, subject verb agreement, spelling acquisition

## Abstract

Children with dyslexia face persistent difficulties in acquiring not only reading skills but also spelling skills. Among difficulties in spelling, problems in grammatical spelling have been studied very rarely. The goal of the study is to better understand grammatical spelling difficulties in children with dyslexia by assessing written syntactic awareness skills, a linguistic factor that has not been investigated in the context of spelling until now. It is worth noting that while morphological awareness has been well studied in children with dyslexia, only very few studies have focused on syntactic awareness, which is, however, necessary to produce number or gender agreement. Twenty children with dyslexia were matched to typically developing children on both chronological age and on grammatical spelling level. All the children were asked to perform a subject verb agreement grammatical spelling test and a written syntactic awareness test on the same sentences, as well as control measures. Results demonstrated that the children with dyslexia performed equally compared to grammatical spelling matched children in grammatical spelling, whilst they performed less well compared to children of the same age. For syntactic awareness, they were less accurate at identifying the subject of the complex sentences than spelling age matched children, even though both groups were matched in grammatical spelling. These results demonstrate that children with dyslexia face a specific deficit in written syntactic awareness. It highlights how better understanding of the spelling difficulty will better guide treatment.

## Introduction

Children with dyslexia who represent between 10 and 15% of school age children (Vellutino et al., 2004) are known to have not only reading but also spelling deficits (Maughan et al., 2009). Spelling deficits include phonetic spelling (phoneme to grapheme correspondences, Angelelli et al., 2004) lexical spelling (spelling of the words, with inconsistent mappings in opaque languages such as French; Alegria and Mousty, 1996) and grammatical spelling difficulties (inflexional suffixes on words, verbs, or adjectives, Egan and Pring, 2004). To date, few studies have evaluated the spelling difficulties of children with dyslexia and even fewer have had the objective of specifically evaluating their grammatical spelling difficulty. However, even for students with dyslexia who have access to higher education, inflectional spelling errors are still observed in text productions and in dictation tasks (Tops et al., 2013). For these reasons, it is important to understand more fully the persistent difficulties encountered by children with dyslexia. In the few studies up to now, some authors have investigated the relationship between grammatical spelling and difficulties in morphological awareness. However, to the best of our knowledge, not one has tried to evaluate a closely related but different skill, written syntactic awareness, which is nevertheless involved in the underlying processes of grammatical spelling.

### Inflectional Spelling in Typically Developing Children

The production of written language, in particular the spelling of words, is described within the framework of computational models. According to Houghton and Zorzi (2003) there are two routes of word spelling: on the one hand, the lexical route, which consists in retrieving the orthographic representations of words stored in the output orthographic lexicon, and on the other hand, the phonological route, which consists in a sub-lexical process of phoneme-grapheme conversion. Spelling new words requires the use of the phonological route, whereas spelling known words or irregular words (i.e., words that contain an infrequent sound-spelling association) requires the use of the lexical route. These processes concern the mechanisms of isolated word production, without integrating the processes involved in the production of sentences or texts.

At the level of the sentence, children have to deal with grammatical spelling rules that are relatively complex depending on the language. While children write isolated words by transcribing what they hear or by retrieving the spelling of words from memory, the production of grammatical spelling is a complex process. Indeed, in some cases, the agreement marks are inaudible, which creates phonemically inconsistent spellings. It requires therefore children to remember to apply the rules. For example, in the French sentence *Les filles regardent le match des garçons* (the girls are watching the boys’ game), the plural agreement on the nouns *filles* (girls) and *garçons* (boys) and the verb *regardent* (are watching) are inaudible. Words in singular (*fille* – girl, *garçon –* boy, regarde – *is watching*) and words in plural (*filles* – girls, *garçons* – boys, *regardent* – are watching) are homophones, which does not allow the plural forms to be written with sound-letter rules. So, children need to know the agreement rules but also they need to know when to apply them. In English, the agreement of regular past verbs presents a similar difficulty for children created by phonemically inconsistent spellings. Indeed, the “ed” endings are pronounced differently depending on the verb, while they are all spelled “ed” (e.g., “kissed” pronounced /kist/ and “killed” as /kild/; Nunes et al., 2006). In order to spell these verbs correctly, children cannot apply the sound-letter rules, they need to learn the grammatical rule of ending for regular past verbs. In Danish, present participle inflection –*ende* is pronounced without *d*, which creates homophones with plural nouns ending with –*ene* (Juul, 2005). For instance, the present participle *legende* (playing) is a homophone of the plural noun *legene* (the games), which requires the mastery of word class differences between verb and noun to choose the correct spelling.

One of the predominant hypotheses concerning the cognitive processes of grammatical spelling acquisition is that of an algorithmic application of the agreement rule, *when I see a subject in plural, I add an –nt mark to the verb* (Fayol et al., 1999; Largy, 2001). This hypothesis of algorithmic application of the rule is interpreted in the theoretical framework *Adaptative Control of Thought* of Anderson (1996) according to which children need to go through three stages in the development and automatization of a cognitive skill: (i) the declarative stage, in which children learn the rules and are able to mention the different steps of their application; (ii) the knowledge compilation stage, in which children start using the different steps (actions); (iii) the procedural stage, in which the rules become progressively automatized thanks to multiple productions requiring the application of the rules. The learning is an attention-demanding process, which will take several months to be mastered and become fast and effortless (Logan, 1988). In French, children usually begin to learn subject verb agreement rules around third grade and are able to manage them around fifth grade (Fayol et al., 1999).

As we have seen, spelling does not only depend on the application of sound-letter conversions, but it requires the ability to apply the grammatical rules. This application of the agreement rules relies in some cases on the mastery of abstract concepts such as the syntactic classes of words. In French, in order to make a verb agreed, children must be able to identify the verb and its subject. In Danish, in order to make a present participle agreed, children must differentiate the present participles from plural nouns. Recognizing the syntactic class to which a word belongs refers to syntactic awareness. Although it is involved in the grammatical agreement process, written syntactic awareness has almost never been studied. Conversely, a related skill, oral morphological awareness has been widely studied in relation to grammatical spelling.

### Morphological Awareness in Typically Developing Children

In an attempt to understand developmental progression in grammatical spelling, several authors have focused on the relationship between grammatical spelling and oral morphological awareness (Nunes et al., 1997a). The relation between awareness of oral language and literacy has been already widely studied at the level of phonological awareness (Bus and van Ijzendoorn, 1999). The studies that will follow focus on the relation between grammatical spelling and awareness of the morphological structure of spoken words.

Morphological awareness is the ability to reflect on and to manipulate morphemes, which are the smallest language units that carry meaning (Nagy et al., 2014). Morphemes within the words can be inflectional affixes (e.g., *assess-ed*) or derivational affixes (e.g., *teach-er*). A wide variety of tasks are used to assess morphological knowledge. The word analogy task has been frequently used in the context of grammatical spelling (Nunes et al., 1997b). The task is entirely oral. It consists of asking the child to transform a word which is analogous to a word that had just been transformed by the experimenter (e.g., *teacher-taught*; *writer* say –*wrote*–). In another morphological awareness task, oral production of verbs is induced by sentence analogy (e.g., *The dog is scratching the chair. The dog scratched the chair. The dog is chasing the cat.* –*The dog chased the cat–*, Nunes et al., 1997b, c, 2006). In other tasks, the instructions are to inflect a pseudoword (e.g., *Say samp. Today the girl samps. What did she do yesterday? Yesterday, she s– samped–*, Walker and Hauerwas, 2006). According to Carlisle (1995) the tasks based on pseudowords are probably more explicit, since children need to manipulate the words and they cannot retrieve the derived word from their lexicon, a process that has been observed in written language (Totereau et al., 1998; Cousin et al., 2002). Through several correlational (Walker and Hauerwas, 2006) and longitudinal studies (Nunes et al., 1997b, c) morphological awareness in spoken language has been shown to be a strong predictor of grammatical spelling performance. Intervention studies have even shown a causal relationship between oral morphological awareness and grammatical spelling (Bryant et al., 1997; Nunes et al., 2003). Morphological awareness interventions were also observed to be particularly beneficial to children with literacy difficulties (Goodwin and Ahn, 2010).

The influence of oral awareness at the morphological level on grammatical spelling skills appears to be demonstrated. However, Egan and Tainturier (2011) showed that orthographic lexical representation was a stronger predictor of past tense spelling than morphological awareness. Therefore, spelling mastery at the lexical level appears to be more related to grammatical spelling than a skill of oral awareness. Egan and Tainturier (2011) understand this less pronounced relationship between spelling and morphological awareness to be because morphological awareness tasks are orally presented and answered by children orally. Morphological awareness tasks also require underlying cognitive processes such as manipulation skills, which are not the same as the processes involved in a written production task.

Consistent with this idea, Juul (2005) suggested assessing the knowledge of grammatical categories through an odd word out written task, in which three of four written words belonged to the same grammatical class (noun, verb or adjective). Danish children had to find the odd one out, for example, find the noun*frakke* (coat) among the three verbs*slippe* (let go), *hente* (fetch), *voelge* (choose). The author found that the knowledge of word classes was correlated to inflectional spelling. This study is interesting because it shows that another kind of knowledge, based on written word class distinctions, appears to be related grammatical spelling.

### Syntactic Awareness in Typically Developing Children

Another aspect of language awareness that has been linked until now to reading abilities rather than to spelling abilities, is syntactic awareness. Tasks were administered in either oral or written modality. Syntactic awareness is the ability to reflect on and manipulate the grammatical well-formedness and syntactic structure of sentences (Bowey, 1986; Cain, 2007; Tong et al., 2014). In Bowey’s first study, syntactic awareness was assessed by an oral error correction task in which the child was informed that the sentence contained a mistake and was asked to say the sentence the right way. However, the intentional manipulation of the syntax was questionable because, according to Gaux and Gombert (1999) it is likely that the child could perform the task based on the semantic violations and an automatic correction of them. In order to create a task that requires a more deliberate manipulation of syntax, Gaux and Gombert (1999) proposed an oral replication task. The task consisted of asking the child to reproduce, in a correct sentence, an agrammaticality presented in an incorrect model sentence (e.g., reproduce a gender agreement error between the article (and adjective) and the noun: *Le (M) dernier (M) voleuse (F) emporte les bijoux*, The last thief is taking away the jewels, in a correct sentence: *Le (M) nouveau (M) coiffeur (M) coupe les cheveux – Le (M) nouveau (M) coiffeuse (F) coupe les cheveux* –, The new hairdresser is cutting hair). In the same task, the authors tested the replication of an incorrect sentence on the basis of an inversion of the word order, for example the inversion of the name and the article. Finally, which is of great interest to the present experiment, they evaluated the identification of a syntactic class of a word within the sentence in the written modality, for example the identification of the subject, verb or adjective, with the same principle of replication. A subject was identified in a model sentence and the child had to underline the word that had the same syntactic function in another sentence. Gaux and Gombert (1999) observed that poor comprehenders exhibited a deficit in the majority of the syntactic awareness tasks.

In sum, the claims of a link between syntactic awareness and written language have so far focused on reading and particularly on reading comprehension. To the best of our knowledge, only one study has considered the relationship between written syntactic awareness and grammatical spelling, by proposing a task of identifying the subject of the sentence. Identifying the subject and checking if it is singular or plural is, however, the first action to be performed in order to execute the algorithmic application of the verbal agreement, according to Anderson (1996). In this first study (Van Reybroeck, 2012) ninety-seven children from grade 4 to grade 6 completed a syntactic awareness task. The authors showed that the task of identifying the subject predicted grammatical spelling performance, after considering variability due to age and to orthographic lexical representation. No study has so far investigated written syntactic awareness in dyslexic children, which could, however, provide new evidence to better understand their difficulties.

### Grammatical Spelling and Morphological Awareness in Children With Dyslexia

Only a few studies have investigated grammatical spelling difficulties amongst children with dyslexia. Their results relate on the one hand to grammatical spelling *per se*, or to the links between this skill and morphological awareness. As far as grammatical spelling is concerned, the authors observed converging results in the direction of a specific difficulty in grammatical spelling. Egan and Pring (2004) demonstrated that children with dyslexia produced more errors on regular past tense verbs in comparison to reading and spelling level matched children. In another study, Hauerwas and Walker (2003) also found with a spelling level matched group of children that difficulties were more pronounced in a sentence context than in a list context, the former being less attention-demanding. In comparing dyslexic children with spelling age matched children, these results support the idea of a specific deficit and a deviant profile rather than a delay in acquisition. Converging evidence comes from the study by Diamanti et al. (2014) conducted in Greek, in which children with dyslexia revealed a delayed more than a deviant performance pattern in spelling derivational and inflectional suffixes, except for verb inflections, where those children performed worse than spelling-level-matched children. For the latter case, they demonstrated a deviant profile. It is important to note that even for students with dyslexia who have access to higher education, grammatical spelling errors are still observed in text production and in dictation tasks (Tops et al., 2013).

In the previous studies (Hauerwas and Walker, 2003; Egan and Pring, 2004; Diamanti et al., 2014) by choosing a spelling level matched group of children as a control group, the authors were able to highlight a deficient pattern in grammatical spelling. They showed that a poorer performance of the dyslexic compared to the matched group was the result of a lower level than expected on the basis of their spelling level. In this way, one should consider that the deficit may not be underpinned by their poorer phonological and orthographic lexical levels. Therefore, why do children with dyslexia have a specific deficit in grammatical spelling? Another explanation can be found by evaluating associated language factors, as studied at the developmental level, which has led some authors to address the question of a possible deficit in oral morphological awareness in children with dyslexia. The evidence for specific difficulties in oral morphological awareness among dyslexic children has been mixed until now. Note that since the aim of the present study was to better understand the development of spelling, the following studies involve children with dyslexia and not children with developmental language disorder, who frequently present syntactic deficits among their deficits in language skills (Bishop and Snowling, 2004).

In one of the first studies, Bryant et al. (1998) found that children who became poor readers initially had a good performance in morphological awareness in word and sentence analogy tasks, but then lost this advantage some time later without being in deficit. Consistent with that view, Egan and Pring (2004) showed that children with dyslexia do not show deficits in morphological awareness in a sentence analogy task, compared with spelling level matched children. Their deficit was limited to reading and spelling. Inconsistent with that view, Hauerwas and Walker (2003) found that children with spelling deficits performed worse than spelling level matched children on an oral morphological awareness task. Thus, these last results suggested a specific deficit in morphological awareness. With regard to the relationship between grammatical spelling and awareness of language, Egan and Tainturier (2011) confirmed that morphological awareness was not a significant predictor of grammatical spelling in children with dyslexia, whereas it was a determinant for typically developing children. Hauerwas and Walker (2003) showed the opposite results since they observed significant relation between grammatical spelling and oral morphological awareness in children with spelling deficits and not in spelling-level matched children.

In sum, it is not clear at present whether children with dyslexia show a specific deficit in oral morphological awareness that could be related to their difficulty in grammatical spelling. It is therefore important to further explore this issue by assessing other factors of language awareness that have not yet been investigated regarding grammatical spelling, such as written syntactic awareness.

### The Present Study

The aim of the study was to better understand the specific difficulties in grammatical spelling encountered by children with dyslexia in evaluating their awareness of language. While several studies have focused on oral morphological awareness, in this study, written syntactic awareness has been evaluated, a linguistic factor that had not been investigated until now with regard to grammatical spelling difficulties. The questions addressed were, first, to know whether children with dyslexia (DYS children) show a deficit pattern in written syntactic awareness using both grammatical spelling level matched children (SL children) and chronological age matched children groups (CA children). The novel aspects of the study were the evaluation of a new linguistic factor, written syntactic awareness, and the use of a specific matching group on grammatical spelling instead of spelling level. Indeed, since lexical spelling and grammatical spelling are based on different underlying cognitive processes, a grammatical spelling match should allow a better understanding of the differences in profiles between children. The second research question was to evaluate the contribution of written syntactic awareness to the variance in grammatical spelling and to look at whether the contribution of syntactic awareness is more or less marked in DYS children than in control children. We made the following predictions: (a) if difficulties in awareness of language may be the consequence of spelling difficulties as argued by Bryant et al. (1998), DYS children should have an equivalent performance in syntactic awareness to SL children; (b) if the difficulties in syntactic awareness are specific and not the consequence of difficulties in spelling, DYS children should perform less well than matched children in grammatical spelling; (c) in the case of a specific deficit in syntactic awareness in DYS children, this skill should contribute to a greater extent to the variance in grammatical spelling in this specific group of children.

## Materials and Methods

### Participants

Sixty-nine French-speaking children from several primary schools took part in the experiment. They were from rural schools in Belgium and were of average socio-economic status. Out of those children, twenty constituted the DYS group (seven girls, 13 boys, *M*_*age*_ = 131.35 months, age range: 113–152 months) and came either from a type eight class of a specialized school in Belgium (specific school and class for students with specific learning disabilities) or from a mainstream school. They had been previously diagnosed with dyslexia by a multidisciplinary team of professionals or by a speech therapist, without having a developmental language disorder. At the time of the study, nineteen of them obtained deficit scores (scores below the 3rd percentile) in the standardized spelling test Corbeau (L2MA, Chevrie-Muller et al., 1997) which is a dictation task in which children’s performance is marked in three areas: phonetic (phoneme to grapheme correspondences), lexical (spelling of the words) and grammatical spelling (agreement rules). One of the twenty children obtained a score close to the deficit threshold: below 4th percentile for a 10-year norm (the oldest norm available), while the child was 12 years old. In reading, children from the DYS group obtained a score below the 4th percentile on the word reading test Lecture en Une Minute (Khomsi, 1998). They also obtained scores below the 16th percentile on the reading comprehension subtest L3 from the Orlec battery (Lobrot, 1967). None of the children were bilingual, according to the criterion of speaking another language for more than 7 h a week (criterion adopted by Marchman et al., 1999).

The DYS children were matched to typically developing children, CA children from Grade 6 (*N* = 24), on the one hand, and on the other hand, to SL children from Grade 4 (*N* = 21). The DYS children were first matched with SL children, typically developing children matched on grammatical spelling level and gender when it was possible (SL children, *N* = 16, nine girls, seven boys, *M*_*age*_ = 119.82 months, age range: 101–130 months). The same dyslexic children were also matched to CA children, typically developing children matched on chronological age, and gender when it was possible (CA children, *N* = 16, eight girls, eight boys, *M*_*age*_ = 136.37 months, age range: 115–148 months). Initially a group of 69 children took part in the experiment but only 52 children were included in the analysis either to allow for a correct match between the groups or because children scored below two standard deviations in the spelling test despite being in the control group. The final groups of typically developing children were composed of 16 children each.

Therefore, the present sample was composed of 52 children. Table 1 provides the characteristics of the participants by group. One-way analyses of variances (ANOVAs) demonstrated an effect of age (*p* < 0.001) and confirmed that the DYS children were correctly matched on chronological age with the CA children. The DYS children were also correctly matched on grammatical spelling with the SL children. All the children’s parents gave their active consent for participation in the experiment and the children gave their verbal consent. The study was approved by the Ethics Committee of the Psychological Sciences Research Institute.

**TABLE 1:** Characteristic of the participants by group: means and standard deviations by group and one-way ANOVA.

		**DYS**		**CA**		**SL**		**Group effects**		***Post hoc* comparisons^a^**
**Measures**		***M***	***SD***	***M***	***SD***	***M***	***SD***	***F***	***p***	
Gender	Girls	15		8		9				
	Boys	5		8		7				
Age in months		131.35	11.27	136.37	12.33	119.82	7.88	10.11	<0.001	SL < CA = DYS
Nonverbal IQ^*b*^		83.06	2.30							
Phonetic spelling	Raw score	8.40	3.50	14.87	0.50	13.19	1.68	36.27	<0.001	DYS < SL = CA
	Standardized score	−5.27	4.63	0.28	0.42	−0.97	1.43	17.29	<0.001	DYS < SL = CA
Lexical spelling	Raw score	7.15	4.49	17.62	3.05	13.31	3.07	36.93	<0.001	DYS < SL < CA
	Standardized score	−3.53	1.34	−0.16	1.15	−1.02	0.91	41.67	<0.001	DYS < SL = CA
Grammatical spelling	Raw score	3.30	1.66	9.50	1.67	4.25	1.61	69.49	<0.001	DYS = SL < CA
	Standardized score	−2.24	1.36	0.15	0.55	−1.64	0.66	27.96	<0.001	DYS = SL < CA
Spelling total	Raw score	18.85	8.63	42.00	4.60	30.75	5.12	55.19	<0.001	DYS < SL < CA
	Standardized score	−3.72	1.92	0.00	0.89	−1.40	0.84	33.98	<0.001	DYS < SL = CA
Reading comprehension	Raw score	14.20	7.32	29.75	4.55	23.31	4.57	32.88	<0.001	DYS < SL < CA
	Standardized score	−1.65	1.16	0.63	0.90	0.66	0.67	35.39	<0.001	DYS < SL = CA

**DYS, Dyslexic children; CA, Chronological age matched children; SL, Grammatical spelling level matched children. ^*a*^Post hoc comparisons are Bonferroni, all p < 0.05. ^*b*^WISC-IV scaled score (Wechsler, 2005; M = 100, SD = 15).**

### Measures

In the experiment, children were administered control measures, used to match DYS children to CA and SL children and to evaluate their written language level, as well as experimental measures, used to answer our research questions.

#### Control Measures

##### Spelling

The standardized spelling test entitled *Le Corbeau* (Chevrie-Muller et al., 1997) consisted of a short text to be written under dictation. The children’s performance was marked on three scores: phonetic (phoneme to grapheme correspondences; maximum score 15), lexical (spelling of the words; max. score 22) and grammatical spelling (agreement rules; max. score 13). The assessment of grammatical spelling was composed of different rules including only two items out of 13 that were verb agreements, the focus of this study. The other items were five past participle agreements, two homophones, one adjective agreement, two noun agreements and one determiner. The maximum accuracy score was 50.

##### Reading comprehension

Reading comprehension skill was evaluated by the standardized subtest L3 from the Orlec battery (Lobrot, 1967). It consisted of a multiple-choice test involving the completion of 36 sentences by selecting the missing word out of five possible options, in a time limit of 5 min. The options included distractors such as homophones (e.g., *mère* [mother] instead of *mer* [sea]), phonological distractors (e.g., *palais* [palace] instead of *balai* [broom]), or semantic distractors (e.g., *pattes* [paws] instead of *oreilles* [ears]). The scores used consisted of the number of words correctly chosen to complete the sentences (max. score 36).

#### Experimental Measures

##### Syntactic awareness

In order to evaluate syntactic awareness, 24 sentences were created. Four types of sentences were created to manipulate the level of syntactic complexity: (a) simple syntactic structure, in which the subject of the sentence directly precedes the verb, e.g., *Les sportifs passent beaucoup de temps dans la salle de musculation* (Athletes spend a lot of time in the weights room); (b) complex noun 1 of noun 2 structure, in which the subject precedes the verb but is distanced by a complex noun phrase, e.g., *Les feuilles de l’arbre tombent dès le mois de septembre* (The leaves of the tree fall from September); (c) complex complement structure, in which the subject precedes the verb but is distanced by a complement of the subject, e.g., *Les débats à propos de la pollution attirent l’attention de tout le monde* (Debates about pollution attract everyone’s attention); (d) interrogative structure, in which the sentence structure is reversed since the subject follows the verb, e.g., *Dans quelle église chantent les choristes pour le concert de Noël?* (In which church do the choir sing for the Christmas concert?). To ensure that the conditions were as similar as possible apart from syntactic complexity, two variables were controlled for verbs: the level of acquisition of lexical spelling from *Echelle d’acquisition en Orthographe Lexicale* (Acquisition scale in lexical spelling, Pothier and Pothier, 2003); and the frequency of the words *Manulex* (Lété et al., 2004). Two Kruskal-Wallis tests revealed no difference between the four types of sentences for the level of acquisition of lexical spelling of the verbs, χ^2^(3, *N* = 24) = 0.08, *p* = 0.99) and for the frequency of the verbs, χ^2^(3, *N* = 24) = 1.31, *p* = 0.73. The sentences and their characteristics are presented in Appendix 1. The children were asked to take their sheet from the grammatical spelling task, and with a green pen circle the word or words that were the subject of the sentence, underline the verb and draw an arrow from the subject to the verb (method inspired by the test used by Aubret and Blanchard, 1991). The children had to perform this task for the sentences they had previously completed for the grammatical spelling task. The internal reliability (Cronbach’s α) in the current sample is 0.95. It is interesting to note that a written, rather than an oral, subject identification task was chosen because when sentences were long, the sentences remained visible, allowing the child to reread them, whereas the same sentences in oral language could be difficult to memorize while performing the subject identification task. The written modality also seemed to be closer to the task that the child is led to do in writing, the purpose of the study being to better understand potential difficulties in grammatical spelling.

##### Grammatical spelling

In order to assess grammatical spelling, the same 24 sentences were presented in a dictation task before the syntactic awareness task. The children had to first listen to the sentences orally, then to write down the two missing words in the blank spaces in the sentences while the sentences were repeated. In order to avoid the children automatically putting plural agreement marks on every verb, verbs with singular agreement were introduced, as well as distractors. So, the task included for example for the simple structure condition, 6 verbs, of which 4 were expected to show plural agreement and 2 singular. Distractors were determinant, preposition or adverb (distractors and verbs in italics in Appendix 1). The internal reliability (Cronbach’s α) in the current sample is 0.94.

### Procedure

All testing took place either at the specialized school or at the mainstream school. For children in the mainstream school, the tasks were conducted collectively in their classrooms in one 40 min session. Some children with dyslexia were assessed in small groups, others were assessed individually, depending on the organization of the class. Children were not informed about the focus on grammatical spelling. To avoid a potential learning effect between the syntactic awareness task and the grammatical spelling task, the children first performed the grammatical spelling task, then the control tasks and finally the syntactic awareness task. Indeed, the identification of the subject within the sentence requested by the syntactic awareness task was likely to help children to better perform the production of the verb agreement in the grammatical spelling task. This is the reason why this test of syntactic awareness was administered in second place. In both parts, grammatical spelling and syntactic awareness, to ensure that the children understood the instructions, an example was given, followed by a training item with individual corrective feedback. To ensure a blind process, the score sheets were anonymized prior to scoring.

### Statistical Analysis

**FIGURE 1:**
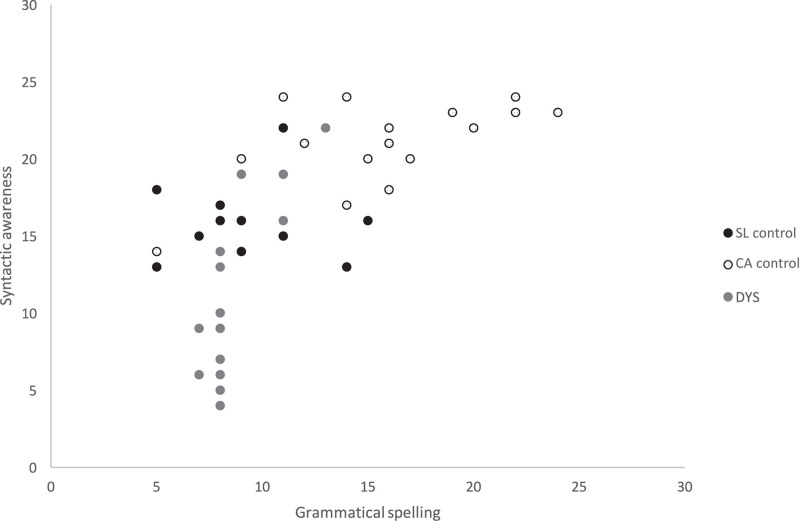
Grammatical spelling and syntactic awareness outcomes by group.

Descriptive statistics for the different variables are displayed in Table 2 and a scatter plot shows in Figure 1 the dispersion of scores for the three groups. The graph shows that the dispersion of scores is particularly important in children with dyslexia for the syntactic awareness task. Statistical analyses were run using SPSS 25. Preliminary analyses were conducted to examine whether the data met the normality assumption of parametric procedures. The analyses revealed no distributional problems (all measures *Sk* <∣2∣ and *Ku* <∣7∣). A Generalized Linear Mixed Model (GLMM) was run instead of a classical analysis of variance in order to include information by item. GLMM was chosen because it allows us to consider the variability of the items and the variability of the participants. Indeed, an analysis of variance does not take into account both the variability introduced by participants and the variability introduced by items in the same analysis, which could possibly lead to high Type 1 error rates (Baayen et al., 2008). GLMM analyses were run on the grammatical spelling and on the syntactic awareness accuracy with Group (three levels: DYS, CA and SL children) and Sentence structure (four levels: simple, complex noun 1 of noun 2, complex complement, interrogative) entered as fixed factors. Furthermore, one random factor was included in the model for participants, allowing us to consider the interdependence between our observations due to repeated measures. The model also included the interaction between Group × Sentence structure. When a main effect was significant, the *post hoc* sequential Bonferroni given by the GLMM is reported. When the interaction was significant, simple effects were analyzed with repeated measures ANOVAs, for which the assumption of sphericity was checked with Mauchly’s test. We applied Greenhouse-Geisser corrections for data violating the sphericity assumption. The alpha level was set at 0.05 for all the analyses.

**TABLE 2:** Means and standard deviations for dependent variables by group.

	**DYS**		**CA**		**SL**		**Group effect**		***Post hoc* comparisons^a^**
**Measures**	***M***	***SE***	***M***	***SE***	***M***	***SE***	***F***	***p***	
**Grammatical spelling**									
Simple structure	0.36	0.05	0.78	0.04	0.39	0.06	23.93	<0.001	DYS = SL < CA
Complex noun 1 of noun 2 structure	0.36	0.05	0.62	0.06	0.39	0.06	6.98	<0.01	DYS = SL < CA
Complex complement structure	0.35	0.05	0.65	0.06	0.33	0.06	11.40	<0.001	DYS = SL < CA
Interrogative structure	0.35	0.05	0.61	0.06	0.37	0.05	5.79	<0.01	DYS = SL < CA
**Syntactic awareness**									
Simple structure	0.83	0.05	1.00	0.00	0.83	0.05	10.81	<0.001	DYS = SL < CA
Complex noun 1 of noun 2 structure	0.62	0.08	0.99	0.01	0.80	0.06	17.01	<0.001	DYS < SL < CA
Complex complement structure	0.22	0.06	0.77	0.07	0.49	0.09	18.41	<0.001	DYS < SL < CA
Interrogative structure	0.12	0.04	0.81	0.06	0.35	0.08	48.14	<0.001	DYS < SL < CA

*DYS, Dyslexic children; CA, chronological age matched children; SL, grammatical spelling level matched children. ^*a*^Post hoc comparisons are sequential Bonferroni, all p < 0.05.*

## Results

### Grammatical Spelling

Grammatical spelling accuracy was submitted to a 3 × 4 GLMM with Group [DYS, CA, SL] × Sentence structure [simple, complex noun 1 of noun 2, complex complement, interrogative] entered as fixed effects. The effect of group was significant, *F*(2, 1227) = 21.94; *p* < 0.001. Sequential Bonferroni *post hoc* showed that the DYS children (*M* = 0.36, *SE* = 0.03) and the SL children (*M* = 0.37, *SE* = 0.04) conjugated fewer verbs correctly than CA children (*M* = 0.67, *SE* = 0.03, each comparison was significant at *p* < 0.01). The effect of sentence structure (*p* = 0.27) and the interaction Group × Sentence structure (*p* = 0.41) were not significant.

### Syntactic Awareness

Syntactic awareness accuracy was submitted to a 3 × 4 GLMM with Group [DYS, CA, SL] × Sentence structure [simple, complex noun 1 of noun 2, complex complement, interrogative] entered as fixed effects. The effect of group was not significant (*p* = 0.09) while the effect of sentence structure was significant, *F*(3, 1235) = 15.77; *p* < 0.001, as well as the interaction between group and sentence structure, *F*(6, 1235) = 2.57; *p* = 0.018. Simple effect analyses showed that the effect of group was significant for the four sentence structures separately: simple, *F*(2, 1235) = 10.81; *p* < 0.001; complex noun 1 of noun 2, *F*(2, 1235) = 17.01; *p* < 0.001; complex complement, *F*(2, 1235) = 18.41; *p* < 0.001; interrogative, *F*(2, 1235) = 48.14; *p* < 0.001. Sequential Bonferroni *post hoc* showed that, for simple structure, DYS children (*M* = 0.83, *SE* = 0.05) identified fewer subjects than CA children (*M* = 1.00, *SE* = 0.00), but the same number as SL children (*M* = 0.83, *SE* = 0.05). For the other three complex structures, DYS children identified fewer subjects than CA but also fewer than SL children. DYS children (*M* = 0.12, *SE* = 0.04) identified the subject of the sentence much less well in interrogative sentences compared to CA children (*M* = 0.81, *SE* = 0.06, *p* < 0.001, *d* = 13.88) and to SL children (*M* = 0.35, *SE* = 0.08, *p* = 0.01, *d* = 3.67; each comparison was significant at *p* < 0.01). For complex noun 1 of noun 2 structure, DYS children (*M* = 0.62, *SE* = 0.08) identified the subject of the sentence less well compared to CA children (*M* = 0.99, *SE* = 0.00) and SL children (*M* = 0.80, *SE* = 0.06). For complex complement structure, DYS children (*M* = 0.22, *SE* = 0.06) also identified fewer subjects than CA children (*M* = 0.77, *SE* = 0.07) and SL children (*M* = 0.49, *SE* = 0.09).

### Factors Associated With Grammatical Spelling

In order to better understand the grammatical spelling profiles of the children, correlations between grammatical spelling, syntactic awareness and the control measures were analyzed. As can been seen in Table 3, the measures were not correlated in the same way in the three groups. In the DYS group, experimental grammatical spelling is most highly correlated with syntactic awareness, *r* = 0.75, *p* < 0.001, while in the CA group, the same correlation is moderate, *r* = 0.60, *p* = 0.01, and in the SL group, it is not significant, *p* = 0.68.

**TABLE 3:** Correlations coefficients between measures by group.

**Measures**		**1**	**2**	**3**	**4**	**5**	**6**
**SL children**							
1	Phonetic spelling	*_*					
2	Lexical spelling	0.40	_				
3	Grammatical spelling	0.47	0.47	_			
4	Spelling total	0.72**	0.88**	0.75**	_		
5	Reading comprehension	0.55*	0.64**	0.79**	0.82**	_	
6	Experimental grammatical spelling	0.25	0.47	0.48	0.52*	0.57*	_
7	Experimental syntactic awareness	0.05	−0.37	−0.13	−0.24	0.07	0.11
**CA children**							
1	Phonetic spelling	*_*					
2	Lexical spelling	0.32	_				
3	Grammatical spelling	0.08	0.76**	_			
4	Spelling total	0.35	0.97**	0.87**	_		
5	Reading comprehension	−0.19	0.50*	0.69**	0.56*	_	
6	Experimental grammatical spelling	0.20	0.83**	0.77**	0.85**	0.37	_
7	Experimental syntactic awareness	−0.05	0.51*	0.57*	0.54*	0.31	0.60*
**DYS children**							
1	Phonetic spelling	*_*					
2	Lexical spelling	0.68**	_				
3	Grammatical spelling	0.59**	0.76**	_			
4	Spelling total	0.87**	0.94**	0.83**	_		
5	Reading comprehension	0.62**	0.81**	0.65**	0.80**	_	
6	Experimental grammatical spelling	0.35	0.71**	0.65**	0.64**	0.67**	_
7	Experimental syntactic awareness	0.38	0.65**	0.71**	0.63**	0.65**	0.75**

*p < 0.05; **p < 0.01.

To evaluate the contribution to the variance in grammatical spelling, hierarchical multiple regression analyses were conducted for each group separately. Phonetic spelling, lexical spelling and syntactic awareness were entered to evaluate their relative importance in predicting grammatical spelling. For all regression models, the collinearity diagnostics showed that all the variance inflation indices (VIF) were below 1.44, indicating that multicollinearity was weak and not a barrier to performing the regression analyses.

As can been seen in Table 4, the regression model accounted for a significant proportion of variance in grammatical spelling in the DYS group, *R*^2^ = 0.67, *p* < 0.001, and in the CA group, *R*^2^ = 0.73, *p* < 0.001, while the model was not significant in the SL group, *p* = 0.19. For DYS children, lexical spelling and syntactic awareness both explained a significant and unique amount of variance in grammatical spelling (lexical spelling: β = 0.54, *p* = 0.03, syntactic awareness: β = 0.47, *p* = 0.02). Conversely, only lexical spelling explained a significant amount of variance in grammatical spelling for CA children (lexical spelling: β = 0.71, *p* < 0.01, syntactic awareness: β = 0.24, *p* = 0.21).

**TABLE 4:** Hierarchical multiple regression analyses of grammatical spelling in each group.

		**DYS**				**CA**				**SL**			
	**Predictors**	***Δ R*^2^**	***B***	***SE***	***β***	***Δ R^2^***	***B***	***SE***	***β***	***Δ R^2^***	***B***	***SE***	***β***
Step 1	Constant	0.12	7.27	0.84		0.04	−14.00	39.29		0.06	3.47	5.58	
	1. Phonetic spelling		0.15	0.09	0.35		2.00	2.64	0.19		0.41	0.42	0.25
Step 2	Constant	0.42**	7.31	0.62		0.65**	1.58	23.51		0.17	2.03	5.32	
	1. Phonetic spelling		−0.10	0.09	−0.25		−0.71	1.65	−0.07		0.12	0.43	0.07
	2. Lexical spelling		0.29	0.07	0.88**		1.40	0.27	0.85***		0.39	0.24	0.44
Step 3	Constant	0.13***	6.52	0.63		0.04**	−11.70	24.98		0.09	−1.59	5.95	
	1. Phonetic spelling		−0.08	0.08	−0.19		−0.15	1.66	−0.01		−0.01	0.43	−0.00
	2. Lexical spelling		0.18	0.08	0.54*		1.17	0.31	0.71**		0.53	0.25	0.59
	3. Syntactic awareness		0.13	0.05	0.47*		0.43	0.33	0.24		0.24	0.19	0.33

*p < 0.05; **p < 0.01; ***p < 0.001.

## Discussion

The aim of the study was to shed light on a factor of language awareness which differs from phonological or morphological awareness, and which has never yet been investigated in relation to grammatical spelling in children with dyslexia. However, syntactic awareness does seem to be one of the underlying cognitive processes related to the application of a grammatical rule. The question was to know: (1) whether children with dyslexia had a specific difficulty in syntactic awareness or not and if so, (2) to what extent that syntactic awareness contributed to variance in grammatical spelling in DYS children compared to in control children. We anticipated that, if DYS children had a specific difficulty in syntactic awareness, they would perform less well than SL children in syntactic awareness. In this case, the contribution of syntactic awareness to grammatical spelling variance should also be greater in this group.

Children with dyslexia and control children were asked to perform a grammatical spelling task and a syntactic awareness task that involved identifying the subject of the sentence and relating it to the verb of the sentence.

### Grammatical Spelling

First of all, the results showed that children with dyslexia have a poorer level of grammatical spelling compared to CA children, which is in accordance with previous studies that present converging results in this direction (Hauerwas and Walker, 2003; Egan and Pring, 2004; Diamanti et al., 2014). This study adds experimental evidence in favor of a grammatical spelling deficit in children learning French, an opaque writing system. Previous studies have been conducted with English-speaking dyslexic children, who also have to learn an opaque writing system (Hauerwas and Walker, 2003; Egan and Pring, 2004) and in Greek, which is a transparent writing system, in Diamanti et al. (2014). In our study, children had difficulty agreeing verbs that contain inaudible agreement marks such as *nt* that constituted phonemically inconsistent spellings. In English, children also had difficulty spelling past tense verbs that contain endings that cannot be written phonetically. In Greek, Diamanti et al. (2014) study showed that, among the difficulties in grammatical spelling, children had more difficulty with verb inflection, also characterized by inconsistencies. Therefore, our study provides an additional argument for a specific difficulty related to verb inflections in children with dyslexia.

The only difference between these studies and ours, apart from the language of the participants, is that we matched dyslexic children with control children on grammatical spelling, while the other authors matched children on reading or spelling levels. By matching children in spelling and demonstrating that dyslexic children are worse, it is possible to argue in favor of a specific grammatical spelling problem (Egan and Tainturier, 2011; Law et al., 2015). In our case, we wanted to match children on grammatical spelling to observe the specificity of their profile in syntactic awareness.

### Syntactic Awareness

Our results provided a first experimental evidence in favor of a specific syntactic awareness difficulty in children with dyslexia. Children with dyslexia showed heterogeneity in their scores on the syntactic awareness task. However, results indicated that they identified fewer subjects of the sentences than both CA and SL children for the three types of complex structure sentences: interrogative, noun 1 of noun 2 and complement. They identified fewer subjects than CA children only for the simple structure sentences. Since the SL children were matched on grammatical spelling level and the dyslexic children faced greater difficulties in finding the subjects of the sentences than them, it is hard to explain how the deficit in syntactic awareness could be caused by a poor level in grammatical spelling. These results lead us to believe that children with dyslexia have a specific difficulty in syntactic awareness and a deviant profile for this language awareness factor.

In order to link these findings to the literature, a broader investigation of studies of morphological awareness is needed, since no studies have examined syntactic awareness in dyslexic children in relation to spelling. This, and the difference in modality between morphological awareness, administered orally, and syntactic awareness, administered in writing, must be taken into account. In this context, our results are in line with those of Hauerwas and Walker (2003) study which showed that children with dyslexia had specific difficulties in morphological awareness in a sentence context, but not in isolated words. In the present study, sentences were also used, which makes it understandable that the results go in the same direction.

On the other hand, the results of our study contradict those of Egan’s study, which showed that dyslexic children do not have a difficulty in the task of oral morphological awareness, whereas difficulties were observed in reading and writing tasks. However, the difference in modality could explain this observation. Indeed, the cognitive processes in an oral task of manipulation of morphemes (Nagy et al., 2014) compared to those of a written identification task are quite different, not only in modality, but also the tasks as such. This could explain why the written syntactic awareness could be altered and not the oral morphological awareness.

### Factors Associated With Grammatical Spelling

Our second research question was to examine to what extent syntactic awareness contributes more to grammatical spelling in children with dyslexia than in control children. The results are also consistent with a specific difficulty of syntactic awareness in children with dyslexia. Indeed, multiples regressions showed that syntactic awareness contributes to variance in grammatical spelling beyond the contribution of lexical spelling, and only in children with dyslexia. In chronological age matched children, lexical spelling is the only factor contributing to grammatical spelling and neither phonetic spelling nor syntactic awareness contribute to it.

Regarding the contribution of syntactic awareness to grammatical spelling, these results are not at first glance in accordance to those of Egan and Tainturier (2011) who found that it is more orthographic lexical memory that makes a contribution to grammatical spelling than morphological awareness. However, the results are not so contradictory because we also observed, like Egan and Tainturier (2011) an impact of the orthographic lexical spelling. In addition, our task of grammatical awareness was in the written and not oral modality, which could cause differences. The proximity between the syntactic awareness task and the grammatical spelling task may allow us to understand this observed link for this specific task and not for the morphological awareness task.

The syntactic awareness task included four types of sentences: a simple type and three complex types in which the subject does not directly follow the verb. Children with dyslexia were less able to identify subjects, even in simple structure sentences. Given that syntactic awareness is a necessary step in achieving grammatical agreement according to Anderson’s model, it can be understood why these children have difficulty with grammatical spelling. While they have to identify nouns that are subjects, or verbs in sentences, they are not clear about these abstracts notions and are not able to implement the different actions required by the rule. It has been shown that cognitive overload related to the management of graphic gesture or lexical spelling can prevent the achievement of grammatical spelling (Van Reybroeck and Hupet, 2009). In the case of children with dyslexia, we know that both graphic gesture (Gosse and Van Reybroeck, 2020) and lexical spelling (Alegria and Mousty, 1996) can be problematic. However, our results lead us to think this: it may not only be cognitive overload that prevents them from producing agreement; it may also be related to their basic mastery of the application of the agreement rules. Moreover, in French, the rule examined in this study is a simple one that requires a basic level of syntactic awareness. After that, the children learn, for example, the rules of agreement of past participles which are more complex (Negro et al., 2014). We can assume that the difficulties of children with dyslexia will be exacerbated for the more complicated rules. One can therefore understand why the difficulties remain persistent even in dyslexic children who are at university (Tops et al., 2013). In this sense, the early identification and assessment of children with difficulties in syntactic awareness is an interesting avenue to investigate.

Regarding the contribution of lexical spelling on grammatical spelling, our results showed that the spelling skills in lexical orthographic representations contribute to the spelling of verb inflections more than syntactic awareness and more than phonetic spelling in both children with dyslexia and chronological age matched children. The contribution of lexical spelling to verb inflections is in line with Egan and Tainturier (2011) study in which the authors showed that the children who have difficulty with grammatical spelling also have a low level of lexical spelling. The influence of lexical spelling level on grammatical spelling was also observed in an experimental study among typically developing children in French. Indeed, Van Reybroeck and Hupet (2009) showed that when words are complex to write at the lexical level, because they are long words or words with inconsistencies, children more often omit the agreement marks in nouns or verbs. The interpretation is that the production of the agreement depends on the cognitive cost of simultaneous processing demands such as the lexical spelling complexity of the words.

Multiple regressions also showed the lack of contribution of phonetic spelling to grammatical spelling. The phonetic spelling represents the assessment of the phonological route according to Houghton and Zorzi (2003) and the sub-lexical process of phoneme-grapheme conversions. The children with dyslexia in our study had a deficient score in phonetic spelling, which means that they made mistakes in basic phoneme-grapheme conversions. Despite this, their level in phonetic spelling did not seem to be related to their level in grammatical spelling. This difference can be understood by the fact that the grammatical spelling does not correspond to the same cognitive processes. Grammatical spelling is a spelling of inconsistencies related to grammatical rules and not to the use of the phonological route. Concerning the mastery of the phonological route, it should also be noted that there is great inter-individual variability in scores in children with dyslexia. It is therefore possible that the level of phonetic spelling influences grammatical spelling for some children and not for others.

### Limitations

This study has several limitations that should guide future research. First, our groups of dyslexic children and control groups were small, which could limit the extent to which the results can be generalized. In particular, the heterogeneity of the profiles of children with dyslexia should lead us to be cautious in generalizing the results to the whole population. It would therefore be interesting to be able to replicate this study with a larger sample. Second, our design study does not allow us to understand the nature of the interactions between syntactic awareness and grammatical spelling. However, it would be interesting to better understand the interactions such as the influence of a low subject identification ability on the learning of verbal agreement or the influence of a limited ability in grammatical spelling on the development of morphosyntactic awareness, given the supposed bidirectional causal relationship between the two skills (Nunes et al., 2006). Third, with regard to the procedure, we have to recognize a limitation because the two tests of grammatical spelling and syntactic awareness, involving the same sentences, were administered on the same day. It is possible that the children read the sentences for the syntactic awareness task more easily, for example, and did not reread them completely. At the same time, their performance on this second task was good for some of the sentences, suggesting that they read the sentences correctly.

### Educational Implications

This study opens up new perspectives on the understanding of grammatical spelling difficulties in children with dyslexia. It highlights the need to evaluate this skill, and work, if required, not only on spelling production but also, prior to that, on understanding and managing the abstract concepts of the nature and function of words that are the basis of the algorithmic application of grammatical rules. In order to be able to apply the algorithm rules, children must be able to actually identify the subject of the sentence before carrying out rule application exercises or activities to automate the processes of written production. A sequence of progressive exercises in which children have to first manage syntactic awareness alone, without adding the cognitive cost of production, is an interesting approach that has been proven to be effective in primary and secondary control children (see intervention studies, Van Reybroeck et al., 2014, 2017). It would be interesting to examine whether children with dyslexia could benefit from this type of treatment based on syntactic awareness.

## Conclusion

The present study has provided, for the first time, experimental evidence in favor of a specific deficit in syntactic awareness in children with dyslexia. Indeed, these dyslexic children were less able to identify the subjects of the sentences, which is required in order to produce verbal agreement. The findings also emphasized the contribution of syntactic awareness to variance in grammatical spelling in children with dyslexia only. These results have important practical implications for teachers and speech therapists in focusing on a new factor of language awareness, syntactic awareness, that may be impaired in children with dyslexia and should be evaluated and trained.

## Data Availability Statement

The datasets generated for this study are available on request to the corresponding author.

## Ethics Statement

The studies involving human participants were reviewed and approved by the Ethics Committee of the Psychological Sciences Research Institute. Written informed consent to participate in this study was provided by the participants’ legal guardian/next of kin.

## Author Contributions

MV contributed to the conception and design of the study, organized the database, performed the statistical analysis, wrote the first draft of the manuscript, and approved the submitted version.

## Conflict of Interest

The author declares that the research was conducted in the absence of any commercial or financial relationships that could be construed as a potential conflict of interest.

## Appendix

**TABLE A1:** List of sentences used in the syntactic awareness task.

**Sentences**	**Verb success rate of spelling in 4th Grade^a^**	**Verb frequency^b^**
**Simple structure**		
Les canards *nagent* tranquillement dans la mare du *parc*	89	60.25
The ducks swim quietly in the pond of the park		
Les sportifs *passent beaucoup* de temps dans la salle de musculation	71	70.37
Athletes spend a lot of time in the weights room		
Les motards *escortent* le cortège présidentiel *depuis* deux heures	75	45.86
Motorcyclists have been escorting the presidential motorcade for 2 h		
Les explorateurs *annoncent* leur *dernière* découverte au journal télévisé	78	61.46
The explorers announce their latest discovery on the news		
Alexandre *cache* ses secrets dans le tiroir de sa *table* de nuit	84	64.15
Alexandre hides his secrets in the drawer of his bedside table		
Le magicien *imagine* un nouveau spectacle *pour* l’année prochaine	91	64.23
The magician imagines a new show for next year		
**Complex noun 1 of noun 2 structure**		
Les perles du collier *brillent* sous *les* projecteurs de la scène	91	60.04
The pearls of the necklace shine under the spotlights of the stage		
Les feuilles de l’arbre *tombent* dès le mois de *septembre*	100	67.57
The leaves of the tree fall from September		
Les aiguilles de l’horloge *tournent* à *nouveau* depuis leur réparation	100	65.26
The clock hands rotate again since their repair		
Les costumes du chanteur *étonnent* un *grand* nombre de spectateurs	58	59.46
The singer’s costumes surprise a large number of spectators		
Le camion des pompiers *retourne* à la caserne *après* l’incendie	75	63.71
The fire truck returns to the fire station after the fire		
L’air *des* montagnes *apporte* beaucoup d’oxygène au corps humain	65	63.05
Mountain air brings a lot of oxygen to the human body		
**Complex complement structure**		
Les débats à propos de la pollution *attirent* l’attention de tout le *monde*	71	59.35
Debates about pollution attract everyone’s attention		
Les inscriptions faites *sur* la porte *dégradent* le bois	79	36.77
The inscriptions on the door degrade the wood		
Ces fleurs rares découvertes il y a un an *poussent* au sommet d*’une* montagne	95	65.27
These rare flowers discovered a year ago grow on the top of a mountain		
Les infirmières dévouées à leur patrie *soignent* ce soldat rapidement et *avec* soin	78	59.01
The nurses dedicated to their country care for this soldier quickly and carefully		
La vie *dans* cette région dévastée par les combats *semble* difficile	71	64.72
Life in this region devastated by the fighting seems difficult		
Le marin affaibli *par* les nombreuses tempêtes *regarde* au loin	90	69.76
The sailor weakened by the many storms looks far away		
**Interrogative structure**		
Dans quelle ville *habitent* tes nouveaux amis rencontrés *en* vacances?	76	63.27
In which city do your new friends you met on holiday live?		
*Dans* quelle église *chantent* les choristes pour le concert de Noël?	88	63.73
In which church do the choir sing for the Christmas concert?		
Combien *coûtent* ces deux pantalons achetés *au* center commercial?	52	56.48
How much do these two pairs of pants bought at the mall cost?		
Dans quelle direction *bougent* les drapeaux décorant la *statue* de la liberté?	96	61.26
In which direction do the flags decorating the Statue of Liberty move?		
Dans quel carton *pleure* mon petit chat *chaque* nuit?	95	62.78
In which box does my little cat cry every night?		
A quelle heure *débute ton* championnat de tennis de table?	67	53.08
What time does your table tennis championship start?		

**^*a*^The success rate of spelling in 4th Grade comes from Echelle d’acquisition en Orthographe Lexicale (Acquisition scale in lexical spelling, Pothier and Pothier, 2003). ^*b*^The verb frequency comes from MANULEX (Lété et al., 2004).**
